# Influence of Surface Roughness on the Mechanical Behavior of Granular Material Under Triaxial Shear: A DEM Study

**DOI:** 10.3390/ma18225251

**Published:** 2025-11-20

**Authors:** Yuqing Tang, Fengyin Liu, Meng Miao, Yu Yin

**Affiliations:** Institute of Geotechnical Engineering, Xi’an University of Technology, Xi’an 710048, China; tangyuqing0139@163.com (Y.T.); miao19950309@163.com (M.M.); 18764209934@163.com (Y.Y.)

**Keywords:** glass beads, surface roughness, discrete element method, shear behavior, meso-mechanism

## Abstract

This study investigates the mechanical behavior of glass bead specimens with different surface roughness under triaxial testing using the Discrete Element Method (DEM). The microscopic parameters for the DEM simulation were calibrated by referencing macroscopic triaxial test data, specifically stress–strain relationships and volumetric changes. We examined the evolution of the contact force network by analyzing the contact forces and the coordination numbers. Our findings indicate that a higher coefficient of interparticle friction results in stronger contact forces, but with a reduced coordination number. A detailed analysis reveals that the strong force network with higher friction, characterized by higher contact forces and a greater density of contacts, becomes more predominant in specimens. Quantitative measures of anisotropy further show that the contact orientations, normal forces, and tangential forces become increasingly anisotropic during shear. These micromechanical findings directly link the enhancement of macroscopic shear strength to the underlying evolution of anisotropic force chains, offering microscopic evidence into the behavior of rough granular materials.

## 1. Introduction

Granular materials are widespread multiphase systems in nature and geotechnical engineering, and their mechanical behavior is crucial for structural stability [1,2]. According to the particle size and the proportion of particle groups, soil can be categorized into coarse-grained soil (sand and gravel) and fine-grained soil (silt and clay), which possess significantly different mechanical properties. The strength of coarse-grained soil is primarily affected by particle characteristics, the density state of the assembly, and stress conditions. The strength of fine-grained soil is determined by its physical state, mineral composition, and stress history. These differences make coarse-grained soils an ideal material for investigating the granular mechanics. The macroscopic mechanical behavior of coarse-grained soil is influenced by various particle characteristics, including size, shape, mineral composition, particle size distribution, and surface morphology [3,4].

Triaxial testing is widely used to investigate these mechanisms, due to its ability to control stress paths and simulate the complex boundary conditions, thereby obtaining comprehensive parameters [5,6]. Wang et al. [7] found through dynamic triaxial tests that increasing the gravel content significantly enhances the liquefaction resistance of gravelly soil. Cho et al. [8] investigated the strength characteristics of natural and crushed sands, revealing that particle irregularity reduces material stiffness but increases its dependence on the stress state. However, the particle characteristics of natural granular materials are complex and interact with each other. Therefore, some studies use glass beads with uniform geometric properties as ideal granular materials to isolate and investigate the influence of individual factors on the shear behavior. For example, Xu et al. [9] conducted a series of drained and undrained triaxial tests on 3 mm diameter glass beads under various confining pressures to investigate their strength, deformation, and critical state behavior. Wu et al. [10] altered the particle size distribution and treated the glass bead surfaces with Teflon to achieve smooth and rough surface properties. They conducted triaxial tests to study the influence of these properties on the shear behavior of the granular assembly. This approach confirms the feasibility of using glass beads as an ideal material for experiments and facilitates direct comparison between numerical and experimental results. In 1979, Cundall and Strack [11] proposed the Discrete Element Method (DEM), which is applicable to discontinuous media. This method overcomes the high cost, heavy workload, and data collection limitations of traditional triaxial tests and has become an effective tool for revealing the macro-micro mechanical mechanisms of granular materials. Some researchers quantified particle shape by defining parameters such as sphericity, angularity, and roughness [12]. The research found that the more irregular the particle shape or the larger the aspect ratio, the higher the shear strength of the granular material [13]. The mechanical properties of soil–rock mixtures, such as the stress–strain relationship and crack development, can be investigated by reconstructing realistic particle shapes through computed tomography (CT) scanning and spherical harmonic analysis, combined with DEM simulations [14,15]. DEM simulation studies have revealed that the microstructure of granular materials significant influences their macroscopic mechanical properties [16]. Moreover, larger particle sizes [17] and wider particle size distributions [18] contribute to higher shear strength in granular materials.

Particularly, particle surface roughness is a key parameter that links surface morphology to the macroscopic mechanical response and plays a significant role in regulating the mechanical behavior of granular assemblies. However, current research mainly focuses on purely simulation methods, which leaves certain research gaps. For instance, Gong et al. [19] used the DEM to investigate the influence of the interparticle friction coefficient (μ) on the critical state friction angle (φ_cs_) of granular materials. They conducted a series of simulated triaxial tests on multi-sphere ellipsoids, and the results demonstrated that φ_cs_ increases with μ, reaching a stable value when μ ≥ 0.3. Antony et al. [20] employed the 3D DEM simulation to investigate the roles of interparticle friction and particle elastic modulus in the shear strength of three-dimensional granular media. They found that the peak shear strength increases with interparticle friction. This study also revealed that both interparticle friction and elastic modulus significantly influence the microscopic structure and macroscopic mechanical behavior of the granular system during shearing. However, these studies lack experimental validation. The synergistic calibration between experiment and simulation is crucial for ensuring result reliability, which would make the findings from DEM simulation research more persuasive. Although some studies have attempted to combine experiment and simulation. For example, Wu et al. [21] conducted triaxial tests to study the shear behavior of homogeneous granular materials and compared the experimental and numerical results. Their validation is still limited to matching macroscopic stress–strain responses. They have failed to establish a multi-scale correlation mechanism linking surface roughness to macroscopic and microscale mechanical behavior.

Building on the research group’s prior investigations of macroscopic mechanics [22,23,24], this study analyzes how the macroscopic evolution of shear strength under different roughness conditions is reflected at the mesoscopic scale. An integrated experimental and numerical approach was adopted. Glass bead specimens with varying surface roughness were prepared and subjected to triaxial consolidated—drained (CD) tests to obtain complete macroscopic mechanical data. Subsequently, numerical simulations of the triaxial consolidation drainage tests were conducted using the DEM software Particle Flow Code in Three Dimensions (PFC3D 6.0). The simulation parameters were calibrated initially based on the stress–strain and volumetric strain, followed by a precise adjustment using the peak strength and friction angle. The mesoscopic mechanisms by which surface roughness influences the shear strength of granular materials were revealed through analyses of coordination number evolution, force chain networks, and contact force anisotropy.

## 2. Experiment Test

### 2.1. Materials

**Figure 1:**
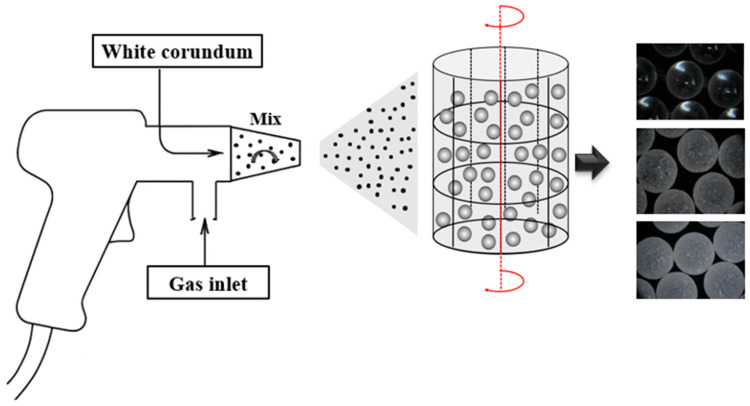
Flowchart of the sandblasting process for the surface of glass beads.

The high-precision borosilicate glass beads (2 mm in diameter), produced by Quanzhou Jinggong Company (Quanzhou City, China) with SiO_2_ as the primary component, were used as the experimental material. Their parameters are listed in Table 1. To prepare glass bead samples with varying surface roughness, a sandblasting treatment was employed. A 60-mesh and a 30-mesh white corundum were used as blasting media. The treated beads, together with untreated ones, form three types of spherical granular materials with different surface roughness levels. These materials are designated as S-1 (no sandblasting), S-2 (60-mesh sandblasting), and S-3 (30-mesh sandblasting). A flowchart of the sandblasting process is shown in Figure 1.

### 2.2. Characterization of Roughness

**Figure 2:**
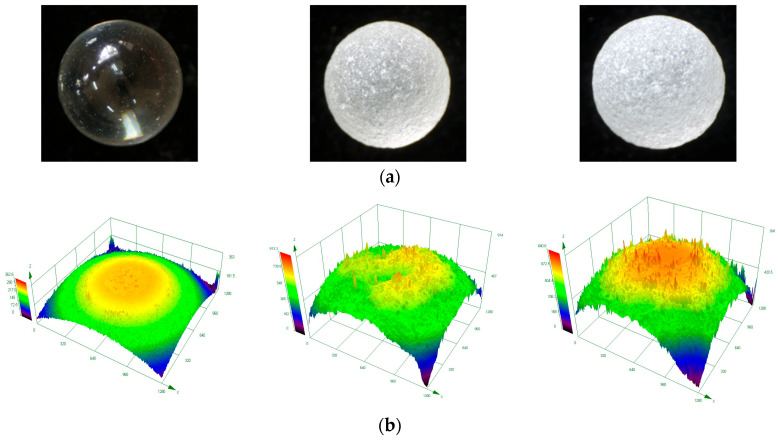
Surface images (**a**) and 3D surface topography (**b**) of the glass beads (from left to right: S-1, S-2, S-3).

The surface roughness of glass beads was measured with LEXT OLS5100 3D laser scanning microscope with its dual confocal system, which integrates both laser confocal optical and color imaging optical systems. The microscope has a lateral resolution of 0.12 μm and uses a laser beam with a diameter of 0.4 μm to scan the sample surface. For the measurements, five samples from each of the three prepared types of glass beads were randomly selected. On each sample, four different locations were randomly chosen. At each location, 20 repeated measurements were taken and averaged for statistical analysis. The measurement area was set to a square region of 1280 μm × 1280 μm. A 10 μm flat area on the sample was selected as the reference surface to minimize curvature-induced errors. The instrument’s built-in software comparison function was used to correct the data, followed by a quantitative evaluation of the surface profile. Figure 2 shows representative surface images and the corresponding 3D surface topography of the glass beads.

According to the Chinese National Standard for Geometrical product specifications (GB/T 33523.2-2017) [25], this study employs three key topography parameters—arithmetic mean height (S_a_), root mean square height (S_q_), and maximum height of the scale-limited surface (S_z_)—to evaluate the three types of rough surfaces. The corresponding measurement results are shown in Table 2. S_a_ is defined as the arithmetic mean of the absolute height values within a defined area, A. S_q_ is the root mean square value of the ordinate values within a defined area, A. S_z_ is the sum of the maximum peak height and the maximum pit depth within a defined area, A. The calculation formulas are given in Equations (1)–(3).

(1) $$S_{q}=\sqrt{\frac{1}{A}\iint_{A}z^{2}(x,y)dxdy}$$

(2) $$S_{a}=\frac{1}{A}\iint_{A}|z(x,y)|dxdy$$

(3) $$S_{z}=Zp_{\max}+Zv_{\max}$$

where A is the measurement area, and z is the height deviation from the reference plane at discrete points. The reference plane is the arithmetic mean height of the surface profile, representing the average height across the entire measurement area. Zp_max_ and Zv_max_ denote the maximum peak height and maximum valley depth, respectively.

As the most widely used parameter in 3D surface topography evaluation, S_a_ characterizes surface roughness, with its value exhibiting a negative correlation with surface smoothness. According to quantitative analysis, the sandblasting treatment significantly increased the surface roughness of the samples. Compared to those of S-1, the S_a_ values of S-2 and S-3 increased by approximately 429% and 679%, respectively. This significant increase in roughness parameters established a foundation for the experiment and provided a reference for calibrating the friction coefficient in subsequent DEM simulations.

### 2.3. Test Program

**Figure 3:**
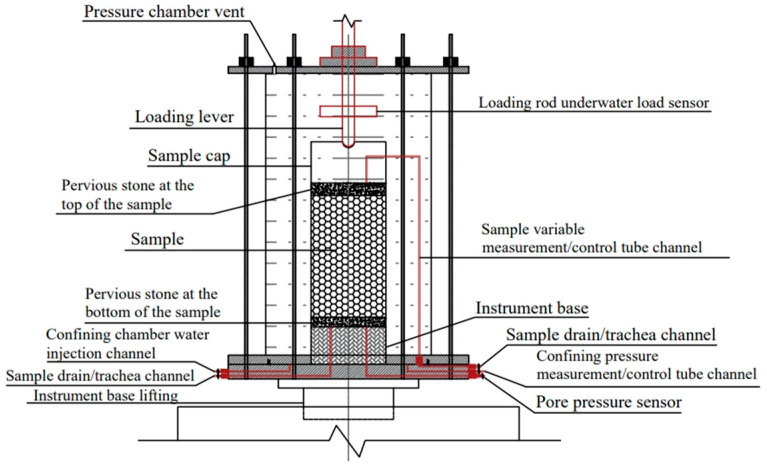
Schematic diagram of triaxial test.

**Figure 4:**
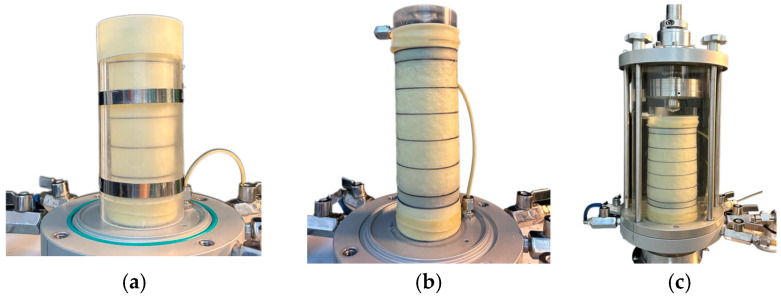
Sample preparation (**a**) installation of the testing mold (**b**) completion of sample preparation (**c**) install the pressure chamber cover.

The experiments used the high-precision triaxial apparatus developed by Contioli Instrument and Equipment Co., Ltd. The schematic diagram of the apparatus is presented in Figure 3. The samples were prepared as cylinders with dimensions of Φ × H = 61.8 mm × 120 mm. Three confining pressures were applied: 100 kPa, 200 kPa, and 300 kPa. The initial void ratio was set to 0.6. Sample was equally divided into five layers and the mass required of each layer was calculated based on the void ratio. The samples were prepared using the layered compaction method to ensure uniform density throughout the sample height. The experimental procedure was as follows:

(1) A 0.5 mm-thick rubber membrane was placed inside the customized membrane-holding cylinder. Then, a vacuum pump was used to extract air from the suction port, causing the membrane to adhere to the cylinder wall. A permeable stone was positioned at the bottom as illustrated in Figure 4a.
(2) The layered compaction method was employed to ensure sample density uniformity. The total sample mass, calculated based on the target void ratio, was divided into five equal portions. Each portion was sequentially placed into the rubber membrane. The surface of each layer was trimmed flush with scale marks on the membrane-holding cylinder using a scraper, following a “Z”-shaped path.
(3) A negative pressure of 10 kPa was applied to the sample interior to maintain its cylindrical shape and prevent collapse after the membrane-holding cylinder was removed as shown in Figure 4b.
(4) Finally, the pressure chamber was assembled, and the securing bolts were tightened to complete the experimental preparation as shown in Figure 4c.

Triaxial consolidated—drained (CD) tests were performed on samples at three confining pressure levels. The confining pressure was applied by filling the pressure chamber with water. The samples were isotropic consolidated (σ_1_ = σ_2_ = σ_3_) for 30 min. The axial load was applied at a displacement rate of 0.24 mm/min. The test was terminated when the axial strain reached 15%. Each confining pressure condition was tested with multiple repetitions, and a new sample was used for each test to ensure the reliability of the results.

## 3. DEM Simulation

### 3.1. Contact Model

This study employed PFC3D 6.0 discrete element software developed by Itasca for the numerical simulation of particle flow. The software provides various contact constitutive models, including the linear model, sliding model, parallel bond model, and Hertz-Mindlin model. The rolling resistance linear model was adopted to accurately characterize the effect of surface roughness on the shear behavior of glass bead samples. Compared to the linear model, the rolling resistance model introduces a rolling friction coefficient (μ_r_) to resist relative rotation between particles. This model provides a more realistic simulation of the energy dissipation mechanism induced by surface roughness. In the experiments, surface roughness was controlled by altering the particle surface morphology through sandblasting. The mechanical effects manifest as changes in the sliding friction coefficient (μ_s_) and the rolling friction coefficient (μ_r_). In the simulations, μ_s_ and μ_r_ were adjusted by a parametric approach to efficiently achieve equivalent simulations of different surface roughness conditions. This approach not only avoids complex surface morphology modeling but also enables precise control of key mechanical parameters.

**Figure 5:**
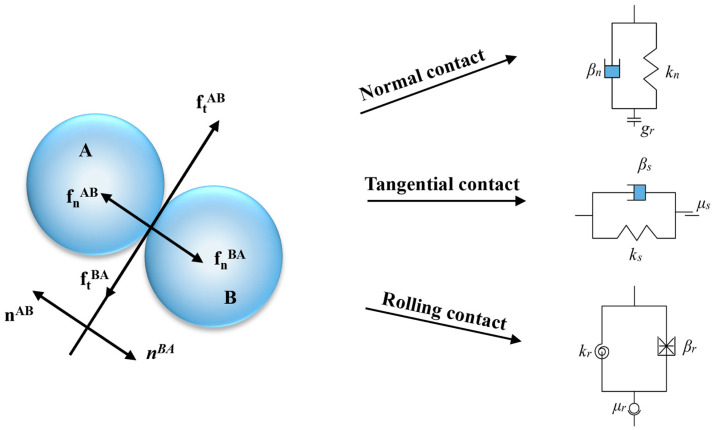
Rolling resistance linear model. (The two particles in contact in the figure are labeled A and B).

As shown in Figure 5, the rolling resistance linear model comprises three components: normal, tangential, and rolling contact models [26,27,28].

The normal and tangential contact forces between particles are computed using Equations (4) and (5).

(4) $$F_{n}=k_{n}U_{n}$$

(5) $$\Delta F_{s}=-k_{s}\Delta U_{s}$$

where F_n_, k_n_, and Un represent the normal contact force, normal contact stiffness, and normal overlap, respectively. ΔF_s_, k_s_, and ΔU_s_ represent the increment of tangential contact force, tangential contact stiffness, and tangential relative displacement, respectively. In the model, the interparticle normal contact stiffness k_n_ and tangential contact stiffness k_s_ can be calculated using Equations (6) and (7).

(6) $$k_{n}=AE/L$$

(7) $$k_{s}=k_{n}/k^{*}$$

where E is the particle elastic modulus. A and L are the cross-sectional area and length at the contact point, respectively. A = πr^2^, r = min (R_1_, R_2_), L = R_1_ + R_2_, where R_1_ and R_2_ are the radius of the adjacent contacting particles. In this study, the samples have a single particle size, therefore r = R_1_ = R_2_, L = 2R_1_ = 2R_2_. k* is the stiffness ratio. When the calculated tangential contact force F_s_ exceeds the maximum static friction μ_s_F_n_, slip occurs between particles, and the contact transitions to sliding contact. The tangential force at the sliding contact is F_s_ = μ_s_F_n_.

The rolling resistance linear contact model is primarily governed by introducing a resisting rolling moment, M_r_, between particles, which is calculated using Equation (8).

(8) $$M_{r}=k_{r}\theta_{r}$$

where θ_r_ is the relative rotation angle between particles. k_r_ is the rotational stiffness, related to the tangential contact stiffness k_s_ and can be calculated using Equation (9).

(9) $$k_{r}=k_{s}{\bar{R}}^{2}$$

where

(10) $$\frac{1}{\bar{R}}=\frac{1}{R_{1}}+\frac{1}{R_{2}}$$

where R is the equivalent radius of contacting particles. When the moment M_r_ between particles exceeds the maximum resisting rolling moment μ_s_RF_n_, rolling occurs, and the contact transitions to rolling contact. The resisting rolling moment at the contact is M_r_ = μ_s_RF_n_.

### 3.2. Parameters in DEM

To ensure accuracy, the parameter ranges were first determined from the literature. The key parameters considered are listed in Table 3. The specific parameter values used in the simulations were determined from the literature and supplier specifications, as detailed in Table 4. This table includes five distinct values of μ_s_ and μ_r_, which were selected to a systematic calibration. The interparticle friction coefficients were calibrated by comparing the numerical and experimental results at identical confining pressures. If the numerical results obtained with selected parameters showed good agreement with experimental results, those parameters were considered calibrated and could be used for subsequent meso-mechanical analysis [21,29].

### 3.3. Test Program in DEM

**Figure 6:**
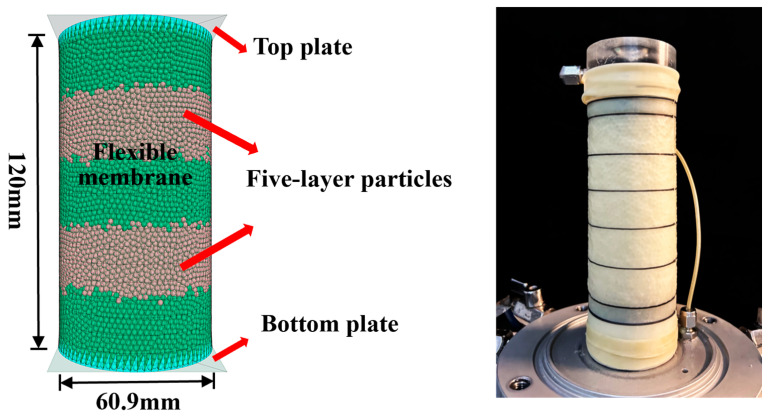
Schematic of the triaxial test preparation process.

The numerical sample comprised a top and a bottom rigid loading platen, a flexible membrane, and a granular assembly, as shown in Figure 6. Rigid wall elements were employed to model the top and bottom loading platens. This approach better captures lateral deformation behavior and facilitates the calculation of volume changes. The simulation process included four stages: sample generation, flexible membrane boundary creation, isotropic consolidation, and shearing.

(1) In the sample generation, the multilayer compaction method was used to generate 53,704 particles within cylindrical rigid boundaries. The sample height was divided into five equal layers. Particles were deposited under gravity. The bottom wall was fixed for each layer, while the top wall position was dynamically adjusted. Interparticle damping and friction were utilized to dissipate residual unbalanced forces below the threshold (set to 1 × 10^−3^) until sample generation was completed.
(2) The rigid wall elements in DEM were replaced with shell elements. The membrane was divided into 81 nodes in the circumferential direction and 20 segments of equal height along the axial direction to balance computational efficiency and membrane flexibility.
(3) The simulation entered the isotropic consolidation.
(4) In the shearing stage, three confining pressures (100 kPa, 200 kPa, and 300 kPa) and the shearing rate (0.24 mm/min) were similarly applied. The test was terminated when the axial strain reached 15%.

The axial stress σ_1_ is calculated as the ratio of the axial force applied to the top platen to the cross-sectional area of the platen. The deviatoric stress and axial strain are calculated using Equations (11) and (12).

(11) $$q=\sigma_{1}-\sigma_{3}$$

(12) $$\varepsilon =\left(h-h_{0}\right)/h_{0}$$

where h is the current height of the sample during shearing, and h_0_ is the initial height of the sample before shearing.

**Figure 7:**
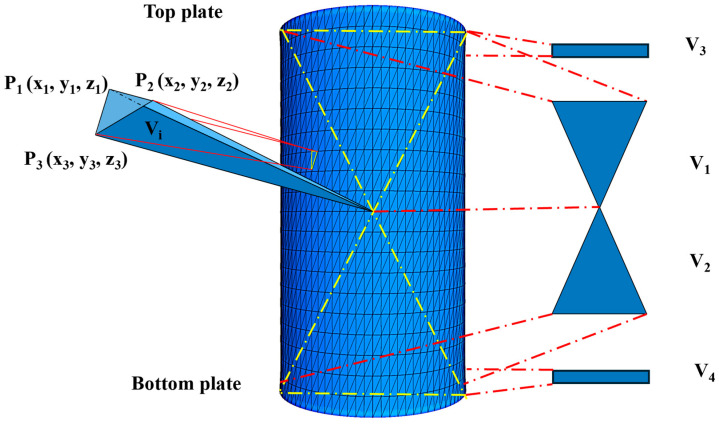
Schematic diagram of the volumetric calculation method.

As shown in Figure 7, to monitor volumetric changes, the real-time volume V of the specimen was calculated by summing five components: V_1_, V_2_, V_3_, V_4_, and ΣV_i_. V_1_ and V_2_ are the volumes of two cones with their apexes at the origin O. V_3_ and V_4_ are the cylindrical volumes resulting from the displacement of the upper and lower loading platens, respectively. ΣV_i_ is the sum of the volumes of tetrahedrons formed by using all the small triangular facets on the sides as bases and the origin O as the apex. Each V_i_ is calculated by the cross product of three vectors formed by the vertices P_1_, P_2_, P_3_ of the triangular facet and the origin O. These volumes are calculated using Equations (13)–(16) [36,37,38].

(13) $$V_{1}=V_{2}=\pi d_{0}^{2}h_{0}/12$$

(14) $$V_{3}=V_{4}=\pi d_{0}^{2}(h_{0}-h)/8$$

(15) $$V_{i}=\frac{1}{6}\left(\begin{array}{ccc} x_{1} & y_{1} & z_{1}\\ x_{2} & y_{2} & z_{2}\\ x_{3} & y_{3} & z_{3} \end{array}\right)$$

(16) $$V=\sum_{i=1}^{n}V_{i}+V_{1}+V_{2}-V_{3}-V_{4}$$

where P_1_ (x_1_, y_1_, z_1_), P_2_ (x_2_, y_2_, z_2_), P_3_ (x_3_, y_3_, z_3_) are the coordinates of the three vertices of the triangular facet. d_0_ and h_0_ are the initial diameter and height before shearing. h is the current height during shearing. n is the number of triangular facets on the lateral surface.

## 4. Results and Discussion

### 4.1. Calibration of Parameters

**Figure 8:**
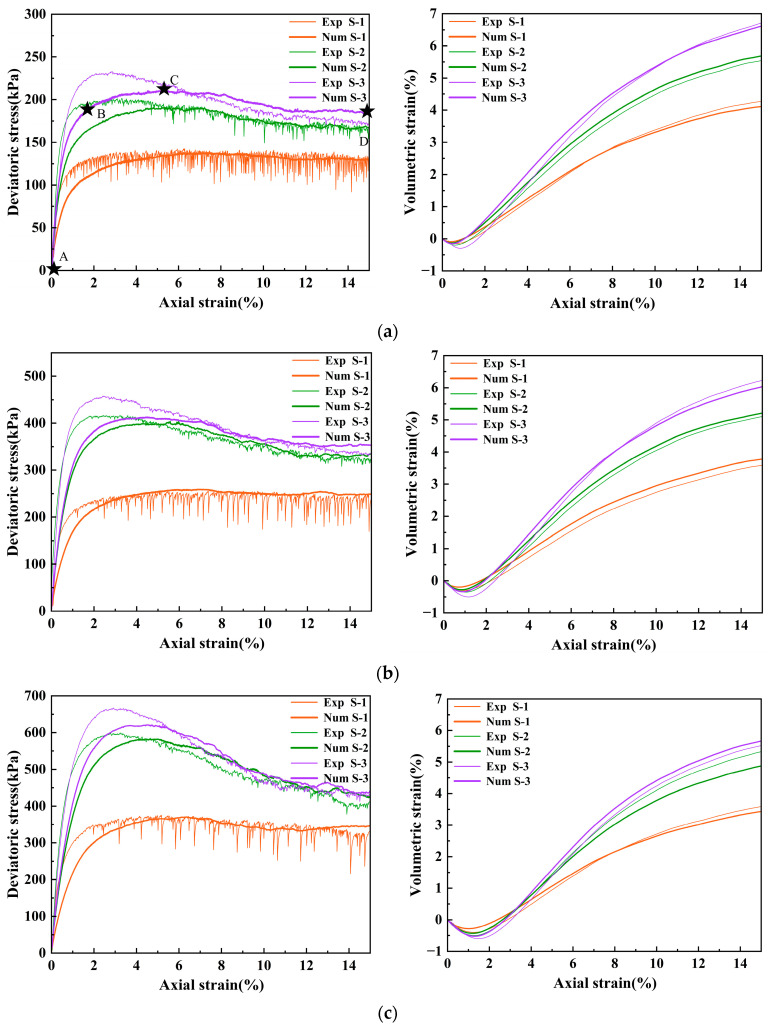
Comparisons of stress–strain and volumetric strain between experimental and numerical results under different confining pressures (**a**) σ_3_ = 100 kPa (**b**) σ_3_ = 200 kPa (**c**) σ_3_ = 300 kPa. (The stars denote the four strain levels (0%, 1%, 5%, and 15%) and are marked as A, B, C, and D in that order).

Figure 8 compares the experimental and numerical results for samples prepared with glass beads of different surface roughness under various confining pressures. As the confining pressure increases, both peak strength and residual strength increase, and strain-softening behavior becomes more pronounced. The peak deviatoric stress of the samples increases significantly with surface roughness which enhances their resistance to shear deformation. Under the same confining pressure, the peak strength increases significantly with the interparticle friction coefficient, and the stress–strain curves transition from strain-hardening to strain-softening. Based on the calibration results, several sets of interparticle friction coefficients were obtained, as shown in Table 5.

**Figure 9:**
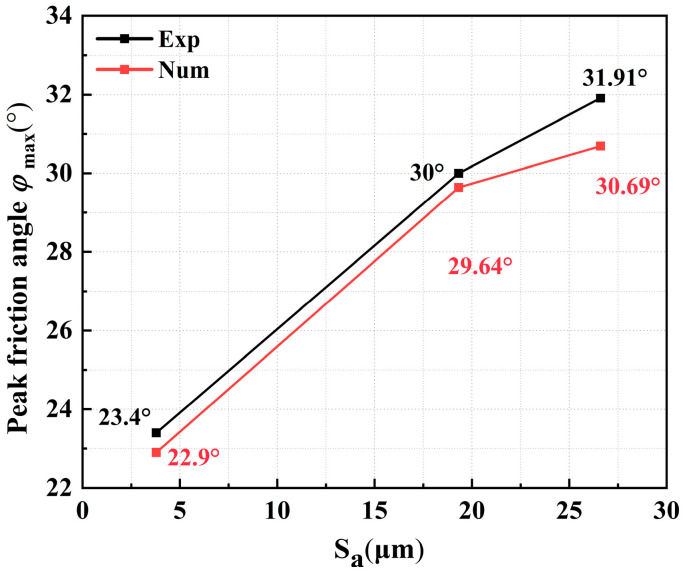
Peak friction angles in experimental and numerical results.

Glass beads with greater surface roughness exhibit higher calibrated interparticle friction coefficients. Correspondingly, experimental and numerical results show high consistency. Statistical analysis of peak strengths from experiments and simulations showed that the maximum relative error is only 9.8%, as shown in Table 6. The absolute error between experimental and numerical values of peak friction angle was less than 1.3°, as shown in Figure 9. Compared to those of S-1, the peak friction angles of S-2 and S-3 specimens increased by approximately 29% and 34%, respectively, and the shear strength improved significantly. The volumetric strain of all specimens shows an initial contractive phase followed by dilation. With increasing surface roughness, the magnitude of dilation increases, and volumetric changes become more pronounced. To investigate the correlation between the strength characteristics of granular materials and the particle surface roughness, the following discussion and analysis are conducted from three mesoscopic aspects: contact force network, coordination number, and anisotropy [22].

### 4.2. Influence of the Interparticle Friction Coefficients on Contact Forces

Force chain networks are the primary pathways for force transmission in granular materials, and changes in their structure directly affect the macroscopic mechanical properties of granular materials. The study divides the total contact network of the granular system into strong and weak contact networks using the average contact force, and quantitatively characterizes them [39,40,41].

**Figure 10:**
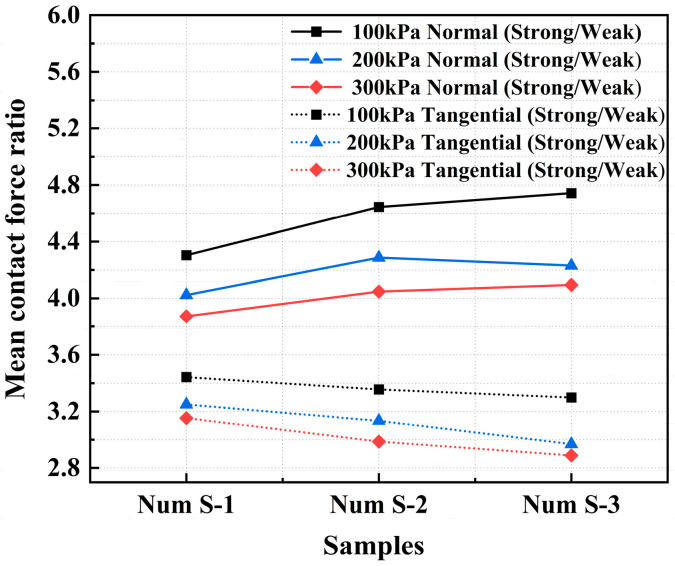
Ratio of the mean contact force in the strong network to that in the weak network versus the interparticle friction coefficient.

Figure 10 shows the variation in the ratios (strong/weak) of both the mean normal contact force and the mean tangential contact force with the interparticle friction coefficient at peak under different confining pressures. As the interparticle friction coefficient increases, the ratio of the mean normal contact forces increases, whereas the ratio of the mean tangential contact forces decreases. This occurs because an increase in the interparticle friction coefficient significantly enhances the mean normal contact force in the strong contact network, whereas the increase in the mean normal contact force in the weak contact network is relatively modest. This differential mechanical response verifies the crucial role of strong contact networks as the primary load-transmission pathway.

**Figure 11:**
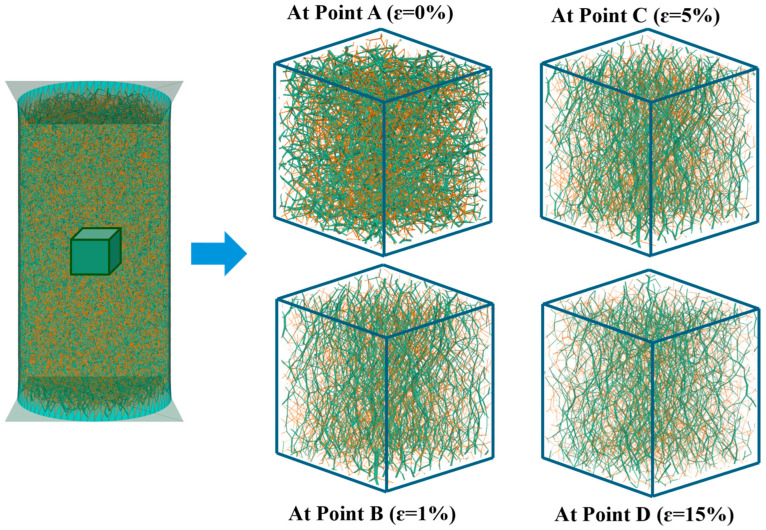
Evolution of the force chain network during the shearing.

Based on the significant differences in mechanical behavior between the strong and weak contact networks, a dynamic analysis of the contact network evolution was conducted during shearing. Using the post-processing capabilities of PFC3D a central cubic region measuring 20 mm × 20 mm × 20 mm was isolated as the representative volume element for analysis. Figure 11 shows the contact force networks at four strain levels (A, B, C, and D) corresponding to the points marked in Figure 8a. Colored cylindrical elements connecting particle centroids represent force chains, where the diameter of each element is proportional to the magnitude of the contact force. Green cylinders represent strong force chains. Orange cylinders represent weak force chains.

The following characteristics were observed in the four networks corresponding to different strain states: At the initial state (Point A), force chains in both strong and weak networks show high directional randomness. The overall network structure exhibits isotropy, with a mean contact force of approximately 0.34 N. With shear advancement to Point B, the weak force chain network remains essentially isotropic, whereas strong force chains progressively orient toward the loading direction. The overall contact network at point B has become noticeably sparser than that at point A, with a mean contact force of 0.63 N. These changes active internal restructuring within the granular assembly. At the peak stress (Point C), the force chain network exhibits a highly anisotropic structure. Vertical strong force chains are substantially reinforced, significantly surpassing those at Point B. The mean contact force reaches its peak value of about 0.75 N. This stage represents the most intensive period of internal structural restructuring, indicating that the primary pathways for stress transmission have been established. At the critical state (Point D), the network is dominated by vertically oriented strong force chains, and the mean contact force stabilizes at around 0.72 N. Internal restructuring achieves a stable configuration. This evolution demonstrates that the strong force chain network serves as the primary conduit for stress transmission. The evolution of strength is governed by normal contact forces [42,43].

**Figure 12:**
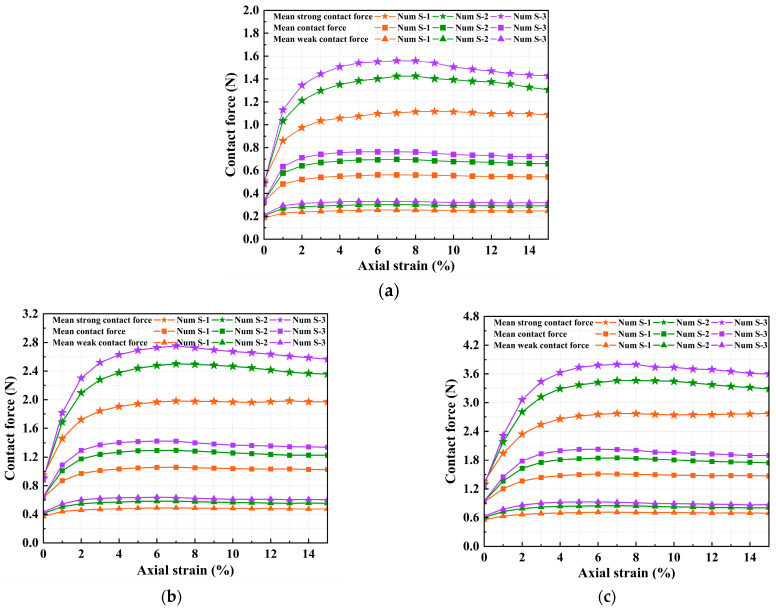
Evolution of contact forces for different interparticle friction coefficients under three confining pressures (**a**) σ_3_ = 100 kPa (**b**) σ_3_ = 200 kPa (**c**) σ_3_ = 300 kPa.

The evolution of contact force chains revealed a progressive strengthening of the strong force network during shearing. To further quantify this, we analyzed the variations in strong and weak contact forces. These variations directly capture the evolving internal stress state. Figure 12 shows the evolution of the mean, strong, and weak contact forces under different interparticle friction coefficients and confining pressures. The magnitudes of all types of contact forces increase significantly with the interparticle friction coefficient. This increase was most pronounced for the strong contact force, which reached a value about four times greater than the weak contact force. This confirms that the strong contact network, acting as the primary load-bearing skeleton, effectively bears the higher contact forces generated by increased particle friction. This originates from the fact that an increased friction coefficient directly leads to greater frictional resistance, thereby promoting contact force enhancement and ultimately resulting in improved shear strength. Furthermore, all contact force types demonstrate varying degrees of enhancement with increasing confining pressure, following consistent evolutionary trends. This provides mesoscopic evidence that increased confining pressure enhances the normal stress between particles.

### 4.3. Influence of the Interparticle Friction Coefficients on the Coordination Number

**Figure 13:**
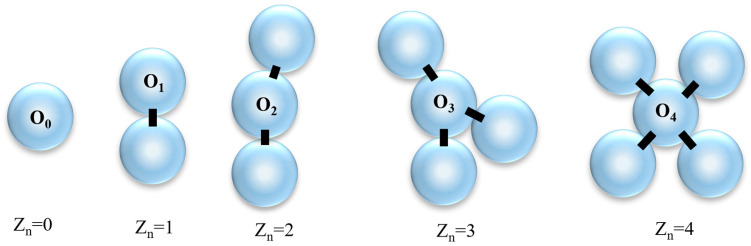
Schematic diagram of particles with different coordination numbers.

The coordination number [12,44], Z_n_, is a dimensionless parameter reflecting the number of interparticle contacts per particle. As shown in Figure 13, let O_n_ denote the set of particles with exactly n contacts. O_0_ (rattlers) do not contribute to force transmission, and O_1_ may represent the “dead ends” of force chains. O_2_ and O_3_ are recognized as the chaining and branching particles, respectively. For n > 3, particles are well stabilized and can exhibit “packed,” “jammed,” and “ordered” configurations [45]. Particles with smaller coordination numbers (excluding O_0_) possess greater degrees of freedom and exhibit higher anisotropy, whereas larger coordination numbers contribute to stability and reduced anisotropy.

The coordination number characterizes the local environment of individual particles. The mean coordination number, ${\bar{Z}}_{n}$, provides a global description of the specimen’s structural characteristics. It is calculated using Equation (17).

(17) $${\bar{Z}}_{n}=\frac{2N_{c}}{N_{p}}$$

where N_c_ and N_p_ are the number of contacts (including particle-particle and particle-flexible membrane contacts) and particles in the specimen, respectively.

**Figure 14:**
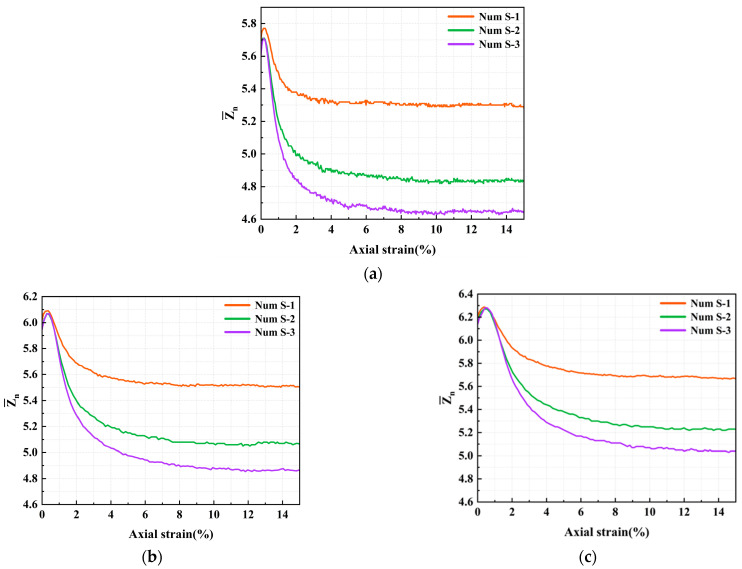
Evolution of the coordination number with axial strain under different confining pressures (**a**) σ_3_ = 100 kPa (**b**) σ_3_ = 200 kPa (**c**) σ_3_ = 300 kPa.

As shown in Figure 14, the evolution of the mean coordination number, ${\bar{Z}}_{n}$, with axial strain under different interparticle friction coefficients is as follows: In the initial small strain stage, ${\bar{Z}}_{n}$ increases due to volumetric compression. As axial strain increases, ${\bar{Z}}_{n}$ undergoes a sharp exponential decline. Eventually, ${\bar{Z}}_{n}$ reaches a stable plateau at approximately 5% axial strain. The higher the interparticle friction coefficient, the lower the stable value of ${\bar{Z}}_{n}$ during the plateau period. This occurs because rougher surfaces increasingly resist particle movement and inhibit the reduction in the number of contacts during shearing. Furthermore, as increasing confining pressure, ${\bar{Z}}_{n}$ increases by about 4%, with consistent evolutionary trends.

**Figure 15:**
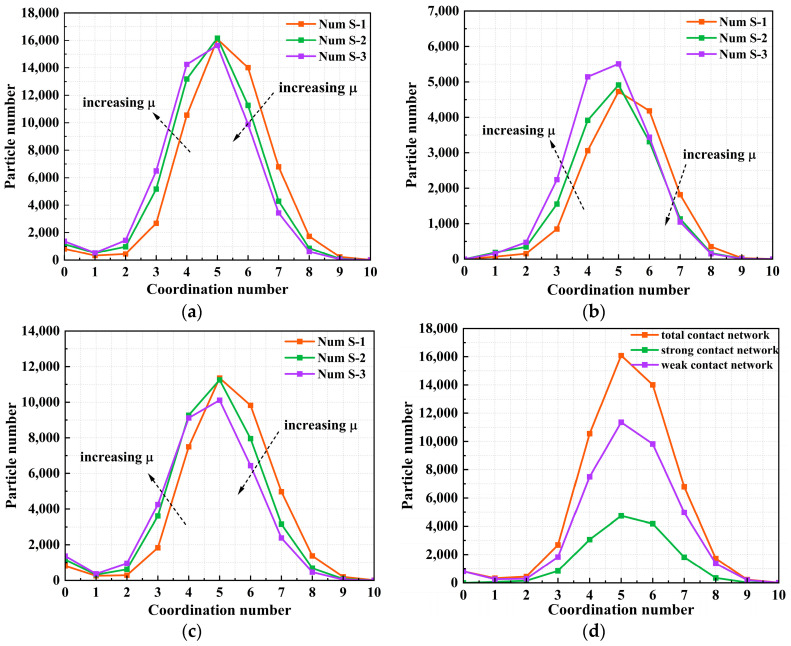
Distribution of particle number versus corresponding coordination number at peak (**a**) Total contact network (**b**) Strong contact network (**c**) Weak contact network (**d**) Num S-1 Total, strong, and weak contact network.

Further analysis was conducted on the coordination number (Z_n_) distribution in the strong, weak, and total contact networks at peak under a confining pressure of 100 kPa, as shown in Figure 15. The mode of coordination number for all contact networks (strong, weak, total) is 5. As friction coefficient increases, the coordination number distribution curves shift leftwards, and the mean coordination number decreases. This leftward shift is especially evident in the strong contact network. This occurs because under the high interparticle friction coefficient, particles cannot easily adjust their positions to maintain stable contacts. This results in the breaking of some originally weaker contacts and the concentration of stress along a few strong contact directions, thereby shifting the distribution toward smaller coordination numbers. A reduced mean coordination number leads to a sparser strong contact network responsible for force transmission, resulting in reduced system stiffness. Load transmission becomes more reliant on a few strong contact points, further reducing the coordination number. This demonstrates that the coordination number in contact networks is significantly influenced by the interparticle friction coefficient. The distribution of particles with different coordination numbers is closely related to the contact characteristics of granular materials. As shown in Figure 15d, the number of particles in the weak contact network is approximately twice that in the strong contact network. This again confirms that the weak contact network primarily provides the basic skeletal structure, which is more stable, whereas the strong contact network acts as the main pathway for stress transmission and exhibits higher dynamic sensitivity during structural reorganization [46,47].

### 4.4. Influence of the Interparticle Friction Coefficients on Fabric Anisotropy

To characterizing stress induced anisotropy in granular systems, a distinction is made between geometric anisotropy and mechanical anisotropy. Geometric anisotropy originates from differences in the local orientation of contact surfaces, resulting in an overall anisotropic. Mechanical anisotropy is primarily induced by external stress and depends largely on contact forces related to the orientation of contact planes. In granular materials, changes in geometric anisotropy can be represented by variations in the contact normal anisotropy parameter a_c_. Contact density rose diagrams provide enhanced visualization of three-dimensional contact anisotropy. A schematic diagram of interparticle contact forces and the contact normal is shown in Figure 5, where n, f_n_, f_t_ represent the contact normal vector, normal contact force vector, and tangential contact force vector between particles, respectively. The fabric tensor formulation proposed by Satake and Oda [20,42,45] is defined by Equation (18).

(18) $$\phi_{\mathrm{ij}}=\frac{1}{N_{c}}\sum_{c=1}^{N_{c}}n_{i}^{c}n_{j}^{c}$$

where N_c_ is the total number of contacts, and n is the unit vector in the direction of the contact plane normal. The probability density of the fabric tensor is calculated using Equation (19).

(19) $$\phi_{\mathrm{ij}}=\int_{\theta }E(\theta )n_{i}n_{j}d\theta$$

where θ characterizes the relative position of n with respect to the global coordinate system. E(θ) is the distribution probability function. The contact normal is commonly characterized by a second-order Fourier expansion of E(θ), and is calculated using Equation (20).

(20) $$E(\theta )=\frac{1}{4\pi }(1+a_{ij}^{c}n_{i}n_{j})$$

where the second-order anisotropy tensor a_ij_^c^ is a deviatoric stress tensor and symmetric, which can be used to characterize the anisotropy of contact directions, as calculated using Equation (21).

(21) $$a_{ij}^{c}=15/2{\phi_{ij}}^{\prime }$$

where φ_ij_^′^ is the deviatoric stress component of φ_ij_.

Variations in mechanical anisotropy can be represented by changes in the normal force anisotropy parameter, a_n_, and the tangential contact force anisotropy parameter, a_t_. Additionally, plotting contact force rose diagrams provides a more intuitive visualization of the evolution of anisotropy within the specimen. For precise definitions of key parameters (a_n_, a_t_), please refer to the literature [42].

**Figure 16:**
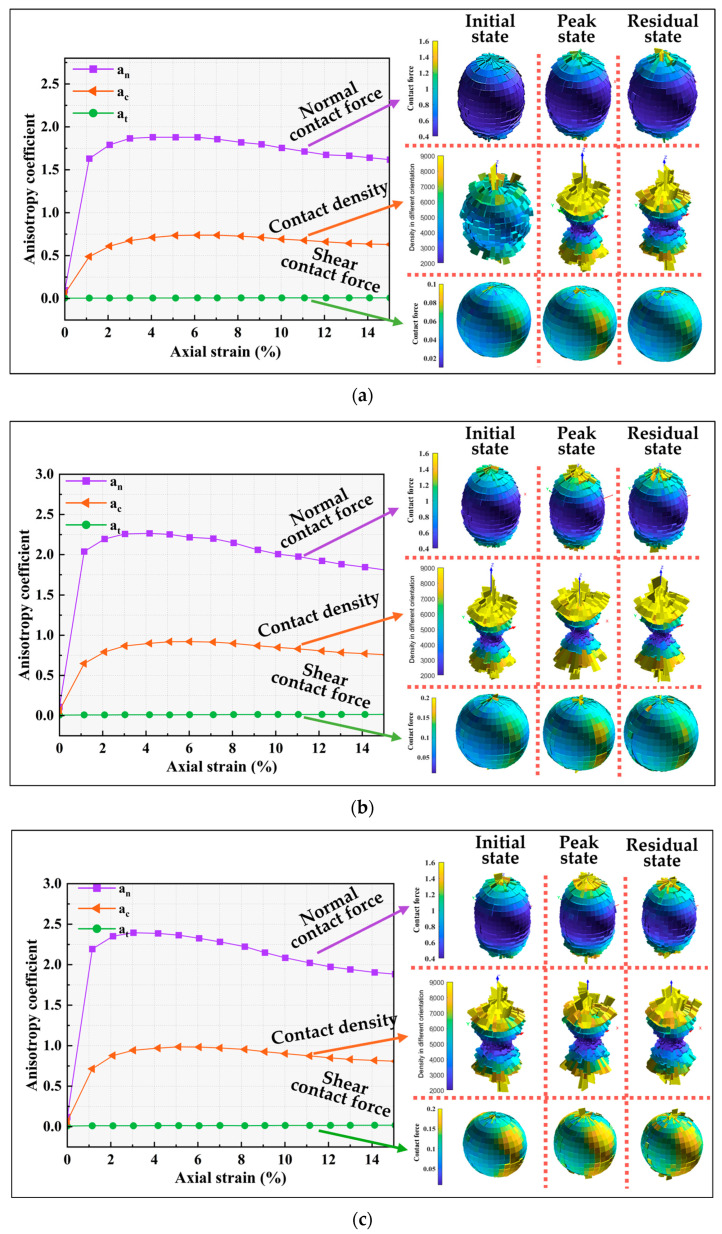

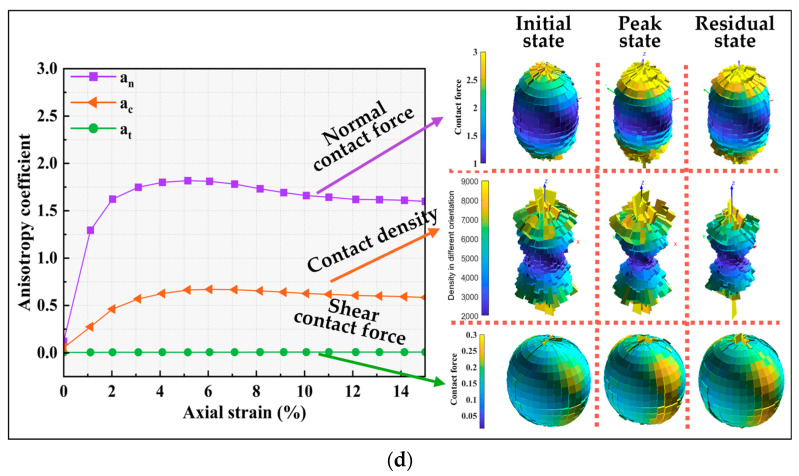
Evolution of anisotropy coefficients with axial strain under different testing conditions (**a**) σ_3_ = 100 kPa Num S-1 (**b**) σ_3_ = 100 kPa Num S-2 (**c**) σ_3_ = 100 kPa Num S-3 (**d**) σ_3_ = 300 kPa Num S-1.

Figure 16 shows the evolution of the anisotropy parameters under various testing conditions, along with spatial distributions of contact density, normal contact forces, and tangential contact forces. Under external loading, the initially isotropic specimens undergo significant internal structural changes. The anisotropy coefficients increase with strain. The spatial distributions indicate that the normal contact forces and contact density undergo more significant reorganization than the tangential contact forces. The contact forces and contact density show marked preferential alignment along the loading axis. Regions closer to the Z-axis exhibit higher contact forces and a greater number of contacts, whereas regions nearer to the XY-plane show lower values. This indicates that larger contact forces are concentrated along the loading direction.

The normal contact force anisotropy parameter a_n_ is higher than a_c_ and a_t_, and its peak occurs earlier. The mechanical response is primarily governed by interparticle normal contact forces, and the development of mechanical anisotropy precedes geometric anisotropy. Comparing Figure 16a,d reveals that the peak values of the anisotropy parameters are larger under lower confining pressure. At the residual state, the anisotropy parameters under different confining pressures tend to converge, demonstrating the limited influence of confining pressure on the developed anisotropy. Meanwhile, an increase in confining pressure significantly enhances the contact forces and the number of contacts, approximately doubling them. Figure 16a–c show that as the interparticle friction coefficient increases, the enhancement of a_n_ is most pronounced. The contact forces and the number of contacts also increase to varying degrees. This suggests that interparticle friction coefficient primarily alters the direction of normal contact forces, thereby improving the shear strength of the specimen [48].

**Figure 17:**
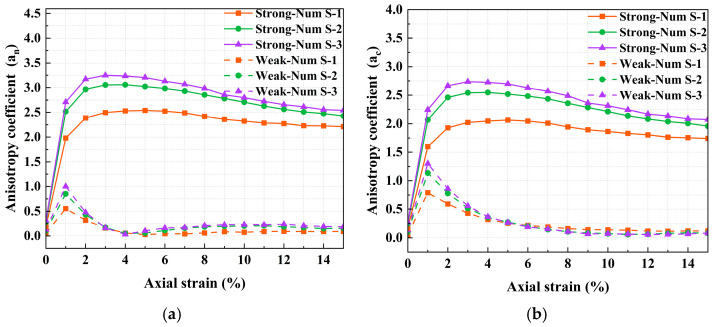
Evolution of anisotropy coefficients with axial strain in the strong and weak contact networks (**a**) contact normal anisotropy (**b**) normal contact force anisotropy.

Figure 17 shows the evolution of contact normal anisotropy and normal contact force anisotropy in strong and weak contact networks. As the interparticle friction coefficient increases, the growth rate of the anisotropy coefficients in the strong contact network increases significantly. This is because higher interparticle friction mobilizes more effectively particles in the strong contact network to resist external forces. Additionally, the values of a_c_ and a_n_ in the strong contact network are greater than those in the weak contact network, and both increase with the interparticle friction coefficient. This indicates that the supporting role of strong contact forces along the loading direction is more pronounced. The anisotropy of the strong contact network contributes significantly to the anisotropy of the total contact network. Similarly to the stress–strain, the peaks all occur at approximately 4% axial strain, corresponding to the attainment of maximum shear strength and peak anisotropy within the strong contact network, which plays a more important role.

## 5. Conclusions

This study investigates the strength characteristics of glass bead specimens in triaxial tests by comparing experimental and numerical results. The effects of strong and weak force chains on the meso-structure of a granular system under varying interparticle friction coefficients were analyzed, focusing on contact network evolution, coordination number changes, and anisotropy development. This study bridges the macro-scale and meso-scale by interpreting macroscopic responses through mesoscopic mechanisms and using macroscopic observations to validate mesoscopic behaviors. The following conclusions can be drawn:

1. Through a comparison of experimental and numerical results, we calibrated the model parameters against stress–strain curves, volumetric strain, and strength data. An increase in the interparticle friction coefficient significantly enhances the shear strength of the specimens.
2. During shearing, the contact force network evolves from an initially uniform distribution to a directional distribution. In this process, strong force chains intensify and orient themselves preferentially in the axial direction. A higher interparticle friction coefficient markedly enhances all contact forces, with the strong force network showing the most pronounced increase. This force drives a more intensive fabric reorganization, which in turn optimizes the pathways for stress transmission This ultimately leads to the enhancement of the macroscopic shear strength.
3. The strong contact network acts as the primary backbone for stress transmission, while the weak contact network maintains structural stability. Under high interparticle friction coefficients, force chains become concentrated due to a reduction in the coordination number. Each contact exhibits stronger tangential friction, which increases the resistance of force chains to breakage under shearing. Strong contact networks enhance shear strength by effectively transmitting shear stress. Interparticle friction controls coordination numbers and mechanical behavior by influencing contact states and the structure of force chains.
4. The contact and force transmission between particles exhibit significant anisotropic characteristics. Mechanical anisotropy develops earlier than geometric anisotropy. Samples with higher interparticle friction coefficient exhibit greater shear strength and anisotropy. Particularly, the anisotropy parameter of normal contact forces is most prominent, and the changes in their spatial distribution are more pronounced. The anisotropy of the normal contact forces within the strong contact network contributes more significantly to the overall mechanical anisotropy.

We acknowledge that this study is bound by its simplifying assumptions. The model ignores particle breakage under high stresses and represents surface roughness indirectly through calibrated friction coefficients, not by direct geometric modeling. To better predict the mechanical behavior of rough granular materials, future work will develop methods for directly modeling surface roughness. Furthermore, coupling these with fluid will be crucial for a more profound exploration of the mechanical mechanisms in unsaturated states.

## Figures and Tables

**Table 1:** Basic parameters of glass beads.

Parameters	Value	Unit
Density	2.5	g/cm^3^
Poisson’s ratio	0.2	-
Young’s modulus	71	GPa
Specific gravity Gs	2.5	g/cm^3^

**Table 2:** The surface roughness index of S-1, S-2 and S-3.

Samples	Roughness Index		
	S_a_ (μm)	S_q_ (μm)	S_z_ (μm)
S-1	3.19	5.17	251.52
S-2	16.91	28.12	739.94
S-3	24.88	52.63	839.75

**Table 3:** Some important parameters in simulation of triaxial test from the literature.

2D/3D	μ_s_	μ_r_	k_n_ (N/m^2^)	k_s_ (N/m^2^)	k*	Damping Coefficient	Literature
3D	0–1	-	-	-	4/3	0.7	[19]
3D	0.15	0.01	-	-	-	0.8	[21]
2D	0.8	0.2	-	-	-	0.3	[27]
3D	0.5	-	4 × 10^5^	4 × 10^5^	1.0	0.7	[30]
2D	0.5	-	1 × 10^8^	1 × 10^8^	1.0	0.05	[31]
2D	0.6	-	4.5 × 10^12^	1.875 × 10^12^	-	-	[32]
3D	0.42	-	1 × 10^7^	1 × 10^7^	-	-	[33]
3D	0.15	0.5	-	-	1.5	0.7	[34]
2D	0.5	0, 0.1, 0.3, 0.5	1.2 × 10^6^	1 × 10^6^	-	-	[35]

**Table 4:** Parameters of simulation.

Parameters	Value	Unit
Sample size (Φ × H)	120 × 60.9	mm
Void ratio (e)	0.61	-
Damping coefficient (d)	0.7	-
Shear Rate (v)	0.04	mm/min
Ball-Ball effective modulus (E*)	1 × 10^8^	N/mm^2^
Ball-Facet effective modulus (E*)	1 × 10^8^	N/mm^2^
Stiffness ratio (k*)	1.5	-
Sliding friction coefficient (μ_s_)	0.1, 0.2, 0.3, 0.4, 0.5	-
Rolling friction coefficient (μ_r_)	0.01, 0.015, 0.02, 0.025, 0.03	-
Shell density/(ρ_m_)	0.93	g/cm^3^
Flexible membrane thickness	0.5	mm
Poisson’s ratio (ν)	0.2	-

**Table 5:** Interparticle friction coefficients.

Samples	μ_s_	μ_r_
Num S-1	0.15	0.015
Num S-2	0.3	0.02
Num S-3	0.4	0.025

**Table 6:** Peak strength in experiments and simulations.

Samples	σ_3_ (kPa)	Exp.	Num.	Relative Error
S-1	100	142.44	138.11	3%
	200	255.86	259.37	1.4%
	300	375.34	370.48	1.3%
S-2	100	201.2	190.84	5.2%
	200	416.47	403.71	3.1%
	300	599.44	582.42	2.8%
S-3	100	232.84	212.38	8.8%
	200	457.75	413.03	9.8%
	300	667.26	620.64	6.9%

## Data Availability

The original contributions presented in this study are included in the article. Further inquiries can be directed to the corresponding author.
